# Cost of Magnetic Resonance Imaging (MRI) and Computed Tomography (CT) scan in UKMMC

**DOI:** 10.1186/1472-6963-12-S1-P11

**Published:** 2012-11-21

**Authors:** Roszita Ibrahim, Sa’don Samian, MZ Mazli, MN Amrizal, Syed Mohamed Aljunid

**Affiliations:** 1 United Nations University- International Institute of Global Health, Kuala Lumpur, Malaysia; 2 Department of Radiology, UKMMC, Kuala Lumpur, Malaysia

## Introduction

Department of Radiology, Universiti Kebangsaan Malaysia Medical Centre (UKMMC), is one of the intermediate cost centres for ward and polyclinic. Magnetic Resonance Imaging (MRI) and Computed Tomography (CT) scan are among the radiology investigations that consume high amount of resources. However, the actual cost of these procedures has never been properly imputed before. The charge for these procedures in UKMMC ranges from RM 550 - RM 800 and RM 100 - RM 450 for MRI and CT scan, respectively. These charges are lower as compared to private sectors and other teaching hospitals in Malaysia. Therefore, the aim of this study is to analyze the cost of MRI and CT scan procedures in UKMMC including the component cost that are involved in these procedures.

## Method

A cross sectional study was conducted from January to March 2012 among radiographer in UKMMC. Activity Base Costing (ABC) method was used to analyze the cost of procedures involving Magnetic Resonance Imaging (MRI) and Computed Tomography (CT) scan. Data was collected using a micro costing form with six component costs which are; 1) staff salary 2) consumables 3) equipments 4) reagents 5) administrative and 6) maintenance of equipment used. The results were analyzed using SPSS version 20.0.

## Results

The cost of MRI procedures with contrast and without contrast per patient was RM 1118.00 and RM 975.00, respectively. Amongst six component costs, equipment was the highest in both with contrast (RM 854.37; 76.4%) and without contrast (RM 854.37; 87.6%) of MRI procedure. While, cost of maintenance (RM 61.51; 6.3%), and staff salary (RM 38.60; 4%) was the lowest in MRI procedures with and without contrast, respectively. For CT scan procedures per patient with contrast for head, orbit and temporal bone was RM 286.00 with the highest component cost was equipment (RM 139.48; 48.8%) and staff salary (RM 35.70; 2.5%) was the lowest. In addition, CT scan procedures per patient with contrast for neck was RM 356.00 with the highest component was equipment (RM 139.48; 39.2%) and staff salary (RM 35.70; 10%) was the lowest. While, the cost of CT scan procedures per patient with contrast for thorax and abdomen was RM 402.00 with the highest component cost was reagent (RM 146.90; 36.5%) and staff salary (RM 35.70; 8.9%) was the lowest. The cost of CT scan procedures per patient without contrast was RM 216.00 with the highest component cost was equipment (RM 139.48; 64.6%) and staff salary (RM 33.15; 15.3%) was the lowest.

## Conclusions

Our data indicate that the cost for the Magnetic Resonance Imaging (MRI) procedure of with and without contrast was RM 1118.00 and RM 975.00, respectively. While, the cost of CT scan procedure with and without contrast was RM 402.00 and RM 216.00, respectively. Cost of equipment was the highest component, thus this finding will contribute to an efficient hospital management in terms of financial and resource used.

